# Translational Regulation of GPx-1 and GPx-4 by the mTOR Pathway

**DOI:** 10.1371/journal.pone.0093472

**Published:** 2014-04-01

**Authors:** Emily N. Reinke, Dede N. Ekoue, Soumen Bera, Nadim Mahmud, Alan M. Diamond

**Affiliations:** 1 Department of Pathology, University of Illinois at Chicago, Chicago, Illinois, United States of America; 2 Department of Medicine, University of Illinois at Chicago, Chicago, Illinois, United States of America; Florida International University, United States of America

## Abstract

Glutathione peroxidase activity was previously determined to be elevated in lymphocytes obtained from patients treated with the Bcr-Abl kinase inhibitor imatinib mesylate. In order to expand upon this observation, the established chronic myelogenous leukemia cell lines KU812 and MEG-01 were treated with imatinib and the effect on several anti-oxidant proteins was determined. The levels of GPx-1 were significantly increased following treatment with imatinib. This increase was not due to altered steady-state mRNA levels, and appeared to be dependent on the expression of Bcr-Abl, as no increases were observed following imatinib treatment of cells that did not express the fusion protein. The nutrient-sensing signaling protein, mammalian target of rapamycin (mTOR), can be activated by Bcr-Abl and its activity regulates the translation of many different proteins. Treatment of those same cells used in the imatinib studies with rapamycin, an inhibitor of mTOR, resulted in elevated GPx-1 and GPx-4 protein levels independent of Bcr-Abl expression. These proteins all belong to the selenoprotein family of peptides that contain the UGA-encoded amino acid selenocysteine. Collectively, these data provide evidence of a novel means of regulating anti-oxidants of the selenoprotein family via the mTOR pathway.

## Introduction

Selenoproteins comprise a family of peptides that contain the essential trace element selenium in the form of the amino acid selenocysteine. During the synthesis of these proteins, selenium is inserted co-translationally in response to one or more in-frame UGA codons, recognized as selenocysteine due to the presence of a Selenocysteine Insertion Sequence (SECIS) element in the 3′ untranslated region of the selenoprotein mRNA [1], [2]. Synthesis of each of the 25 human selenoproteins is controlled by common mechanisms requiring a dedicated tRNA, tRNA^[Ser]Sec^, additional elongation factors and accessory proteins for their translation, as well as regulatory mechanisms that are unique to each member of the selenoprotein family [3]. The synthesis of several of these selenoproteins is responsive to selenium availability, and the mechanisms by which this regulation occurs include nonsense mediated decay of selenoprotein mRNAs due to the presence of in-frame UGA codons [4], [5] and the interaction between the SECIS binding protein 2 and eukaryotic initiation factor 4a3 [6].

GPx-1 was the first characterized selenoprotein and functions to detoxify hydrogen and lipid peroxides using reducing equivalents from glutathione, impacting reactive oxygen mediated signaling in addition to the more apparent role as an anti-oxidant (see reference [7] for a comprehensive review). GPx-1 enzyme activity may be modified post-translationally, with there being evidence that acetylation [8], [9] and phosphorylation [10] are among the processes that might impact activity. In the case of the latter, GPx-1 has been reported to be phosphorylated by the c-Abl and Arg non-receptor tyrosine kinases, resulting in the enhancement of activity [10].

Based on the observation that GPx-1 activity could be enhanced by tyrosine phosphorylation and inhibited using the Bcr-Abl small molecule inhibitor imatinib [10], we explored the possibility that patients being treated for chronic myelogenous leukemia (CML), a form of leukemia characterized by the Bcr-Abl translocation and treated with imatinib, would possess mononuclear blood cells with decreased GPx activity. Contrary to expectation, most of the samples examined from CML patients expressed increased GPx enzyme activity following imatinib therapy [11]. To examine this phenomenon in greater detail, studies were initiated in which established CML cell lines were exposed to imatinib in culture and consequential effects on GPx-1 examined.

## Materials and Methods

### Cells and culturing conditions

KU812 and MEG-01 human CML cell lines and MDA-MB-231 and MCF-7 human breast cancer cells were obtained from ATCC (Manassas, VA). The GM10832 human immortal B lymphocyte cell line was obtained from the National Institute of General Medical Sciences Human Genetic Cell Repository of the Coriell Institute (Camden, NJ). All cells except MCF-7 were maintained in RPMI-1640 (ATCC). MCF-7 cells were maintained in MEM (Gibco, Grand Island, NY). All media was supplemented with 10% fetal bovine serum (FBS, Gemini Biosciences, West Sacramento, CA), 100 units/ml penicillin, and 100 μg/ml streptomycin (Gibco). A sub-clone of KU812 cells was obtained subsequent to methylcellulose colony formation in MethoCult (Stemcell Technologies, Vancouver, BC). Visible colonies were isolated after 14 days of incubation and expanded. For all pharmacological treatments, non-adherent cells (KU812, MEG-01 and GM10832) were suspended at 2×10^5^ cells/ml and treated with 150–300 nM imatinib mesylate (Novartis, Basel, Switzerland) for 3–7 days, 1 ng/ml rapamycin (Cayman Chemical, Ann Arbor, MI) for 3 days or 20 μM LY294002 (Cayman) for 4 days. For 7 day imatinib mesylate exposures, fresh media and imatinib were applied on day 3. For protein half-life studies, KU812 were also exposed to 25 μg/ml cycloheximide for 3 days. MDA-MB-231 and MCF-7 cells were plated 1 day prior to treatment with imatinib or rapamycin. Fresh media was applied and cells were treated with 500 nM imatinib for 7 days or 1 ng/ml rapamycin for 3 days. As with the 7 day treatment of non-adherent cells, fresh media and imatinib were applied on day 3. Cells were maintained at 37°C and 5% CO_2_ in a humidified incubator.

The retroviral Bcr-Abl expression construct, which expresses green fluorescence protein (GFP), was generously provided by Dr. WenYong Chen [12]. MDA-MB-231 cells were transfected with empty vector, Bcr-Abl, and kinase-deficient Bcr-Abl with X-tremeGENE HP DNA transfection reagent (Roche Applied Science, Indianopolic, IN). Cells were co-transfected with pSV2-hph (ATCC) at a 1∶40 molar ratio for hygromycin B (Cellgro, Manassas, VA) selection. GFP-positive colonies were detected by fluorescence microscopy and isolated for expansion.

GP293 retroviral packaging cells (Clontech, Mountain View, CA) were maintained in high-glucose Dulbecco’s Modified Eagle Medium (Gibco) with 10% FBS, 100 units/mL penicillin and 100 μg/mL streptomycin. GP293 were co-transfected with the pVSV-G pantrophic packaging vector (Clontech) and pLNCX-UGA or pLNCX-UGA-GPx1 SECIS reporter constructs [13], [14] using Lipofectamine (Invitrogen). Forty-eight hours after transfection, virus containing supernatant was filtered, 4 μg/mL polybrene (Santa Cruz Biotechnologies, Santa Cruz, CA) added to supernatant (multiplicity of infection > 1×10^6^) and 1 mL was applied to MDA-MB-231 cells on 35-mm tissue culture plates. Infection proceeded for 6 hours, at which point an additional 1 mL of RPMI-1640 containing 4 μg/mL polybrene was added and cultures were incubated for an additional 18 hours at which time the infection media was removed and fresh RPMI-1640 applied, and where indicated, cells were treated with 1 ng/mL rapamycin.

### Protein, RNA and enzyme activity analyses

Protein samples were prepared for western blotting in NuPAGE LDS sample buffer (Invitrogen, Grand Island, NY) with NuPAGE sample-reducing agent, boiled for 10 minutes, electrophoresed on a 4–12% Bis-Tris denaturing polyacrylamide gel (Invitrogen) and transferred to PVDF membranes (Millipore). Antibodies against the following proteins were used: GPx-1 (mouse, MBL, International, Woburn, MA), MnSOD (mouse, BD Biosciences, San Jose, CA), GPx-4 (rabbit, Abcam), TrxR1 (rabbit, Proteintech, Inc., Chicago, IL), phospho-S6 (rabbit, pS6, Cell Signaling), and β-actin (rabbit, Abcam). Protein bands were quantified using ImageJ software (NIH, Washington, DC) and normalized to β-actin band density. Total GPx enzyme activity was analyzed by a coupled spectrophotometric GPx assay, as previously described [15].

RNA was isolated from treated cells using the RNeasy RNA isolation kit (Qiagen, Valencia, CA), and quantified by NanoDrop (Thermo Scientific, Wilmington, DE). Two μg of total RNA in a 40-μL reaction was reverse-transcribed with the High Capacity cDNA Reverse Transcription kit following the manufacturer’s instructions (Applied Biosystems, Grand Island, NY). Primer-probe pairs were obtained from Applied Biosystems for GPx-1, MnSOD, TATA-binding protein (TBP), and 18S RNA. Expression was analyzed in a 96-well plate format using a Step–One Plus Instrument (Applied Biosystems) with the following protocol: 50°C for 2 minutes, 95°C for 10 minutes, followed by 40 cycles of 15 seconds at 95°C and 1 minute at 60°C. The cycle threshold (Ct) value was determined, and changes in expression relative to untreated controls were calculated using the2^−ΔΔCt^method [16]. The Ct values for GPx-1 and MnSOD were normalized to the Ct value of selected housekeeping genes. Three independent cultures were analyzed in triplicate for each treatment condition.

### Construction of plasmids

GPx-1 expression constructs were previously generated [17]. A derivative GPx-1 mutant designated U49C was generated by *in vitro* mutagenesis. The mutation results in the alteration of codon 49 of GPx-1 from a Sec (U) to a Cys (C) utilizing the QuickChange II Site-Directed Mutagenesis Kit (Agilent Technologies, Santa Clara, CA). In brief, template DNA was amplified utilizing two overlapping primers (forward primer, 5′-GTGGCGTCCCTCTGTGGCACCACGGTC-3′; reverse primer, 5′-GACCGTGGTGCCACAGAGGGACGCCAC- 3′) and incubated with *Dpn*I to digest the original template DNA. Mutation of the TGT codon was verified by sequencing at the UIC DNA Services facility. A clonal population of MDA-MB-231 was transfected for 4 hours with the U49C construct using Lipofectamine 2000 (Invitrogen); fresh RPMI-1640 was applied following transfection. Transfected cells were selected with 500 μg/ml G418 and surviving colonies were isolated by trypsin and cloning rings. Individual clones with baseline GPx activity and protein levels (no different from vector only controls) were expanded for analysis. Cells were treated with 1 ng/ml rapamycin for 3 days and GPx enzyme activity as well as GPx-1 and actin protein levels were determined.

## Results

### Imatinib increases GPx-1 enzyme activity and protein levels in Bcr-Abl expressing cells

**Figure 1:**
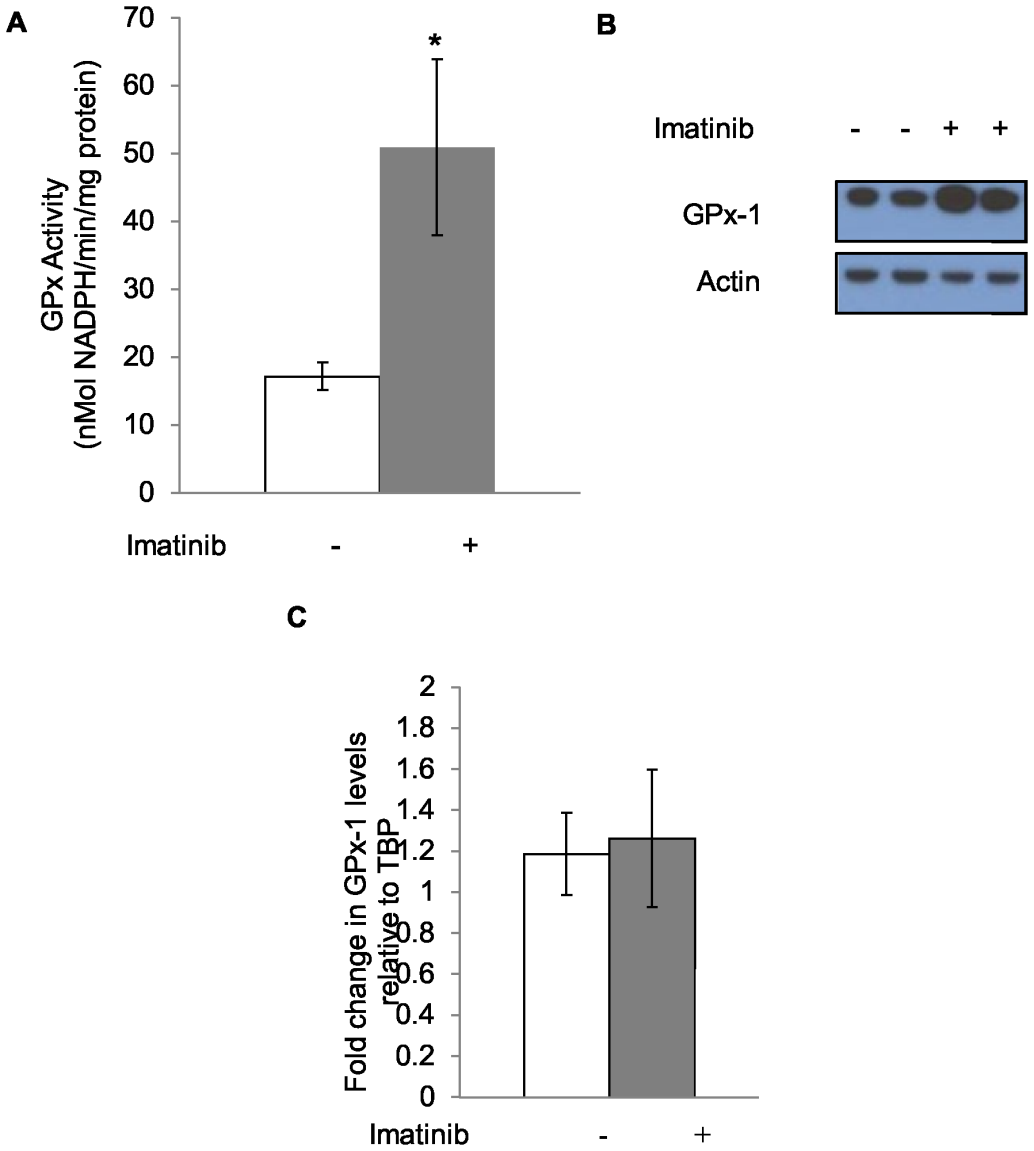
GPx-1 activity and protein, but not steady-state transcript levels are enhanced by treatment with imatinib. KU812a cells were treated for 7 days with 150 nM imatinib and the effects on GPx enzyme activity (A), GPx-1 protein (B) and GPx-1 transcript levels expressed as the GPx-1 Ct values normalized to the Ct values for TBP mRNA(C) is shown. GPx activity was increased 2.5-fold, and protein increased 7-fold (*P* = 0.02) by imatinib treatment, while steady-state transcript levels were unaltered. Data shown in A and C are the results of three independent experiments. * = *P*<0.001. Error bars indicate the S.D.

**Figure 2:**
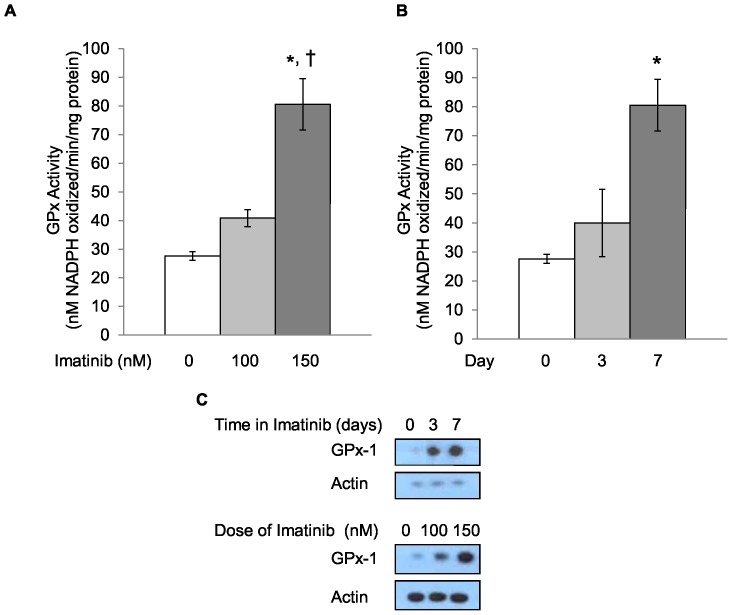
GPx-1 levels are enhanced in a dose- and time-dependent manner following imatinib treatment. The dose- and time-dependent increases in GPx activity following 100 nM and 150 nM imatinib treatment of KU812a cells for 7 days (A) or 150 nM imatinib treatment for 3 or 7 days (B). The effect of imatinib treatment on GPx1 protein levels. Data shown is representative of thee independent experiments were performed (C). * = *P*<0.001, † = *P*<0.05, compared to 100 nM or 3-day imatinib treatment. Error bars indicate S.D.

**Figure 3:**
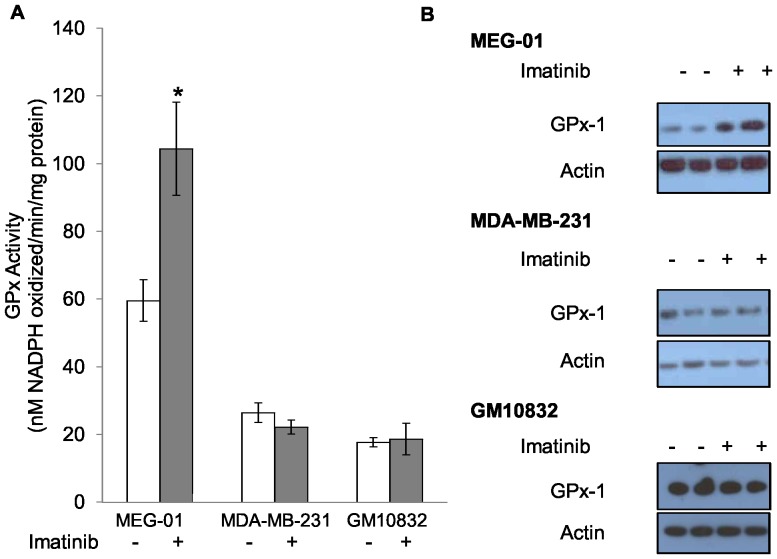
GPx-1 is increased in MEG-01, but not MDA-MB-231 and GM10832, following imatinib treatment. The effect of imatinib on GPx enzyme activity (A) and GPx-1 protein (B) in MEG-01, MDA-MB-231 and GM10832 is shown. GPx activity was increased 2-fold, and protein increased 4-fold (*P*<0.01) by 300 nM imatinib treatment in MEG-01. GPx-1 activity and protein levels did not change following imatinib treatment of MDA-MB-231 (500 nM imatinib) and GM10832 (300 nM). Data presented in A is the result of three independent experiments * = *P*<0.001. Error bars indicate S.D.

In order to determine if GPx activity is altered by imatinib treatment *in vitro*, a subclone of KU812 human CML cells (KU812a) was exposed to a cytostatic dose of 150 nM imatinib for 7 days. GPx activity levels were increased 2.5-fold from baseline levels seen without treatment (Fig. 1A). The increase in activity was not as great as that observed for the protein levels as determined by western blotting with anti-GPx-1 antibodies, which increased 7-fold from baseline (Fig. 1B). A time course and dose response for the imatinib effect was determined for KU812a cells by treatment with imatinib at 100 or 150 nM imatinib for 3 to 7 days (Fig. 2A-C). The stimulation in GPx-1 was not associated with an increase in the steady state levels of GPx-1 mRNA as determined by quantification of GPx-1 mRNA levels by RT-qPCR of mRNA obtained from imatinib-treated and untreated KU812a (Fig. 1C). Similarly, imatinib did not appear to alter the stability of GPx-1 as treatment of KU812a cells with cycloheximide (25 μg/ml) and/or 150 nM imatinib for 72–96 hours remained at approximately 72 hours (data not shown). The effect of imatinib on GPx-1 was not limited to KU812 cells. Incubation of another CML cell line, MEG-01 [18] with a cytostatic dose of imatinib (300 nM) for 7 days resulted in a 1.6 fold increase in activity (Fig. 3A) and a 4 fold increase in GPx-1 protein levels (Fig. 3B).

**Figure 4:**
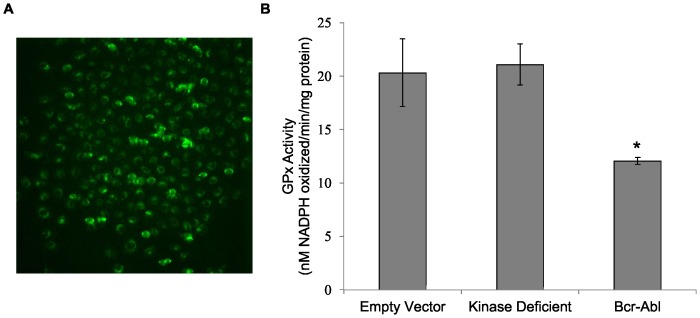
GPx-1 protein and activity levels are decreased by exogenous Bcr-Abl in MDA-MB-231. MDA-MB-231 cells transfected with a GFP-tagged Bcr-Abl vector and the transfection success was as assessed by fluorescence microscopy (A). The effect of ectopic Bcr-Abl expression on GPx enzyme activity in MDA-MB-231 cells. GPx activity was decreased 0.6-fold by ectopic expression of Bcr-Abl. Data shown is representative of one experiment. Two independent experiments were performed. * = *P*<0.001. Error bars indicate S.D.

In contrast to the data obtained with CML cells, imatinib did not stimulate GPx-1 levels or activity in non-Bcr-Abl expressing human cell lines. The immortalized lymphocyte cell line GM10832 and breast cancer-derived cell line MDA-MB-231 were each exposed to cytostatic doses of imatinib (300 nm and 500 nM, respectively) for 7 days and no significant change in either GPx activity or GPx-1 protein levels were observed (Figs. 3A and 3B). To further investigate the role of Bcr-Abl in the regulation of GPx-1, MDA-MB-231 cells that do not express the Bcr-Abl fusion protein were transfected with either an empty vector, a Bcr-Abl expression construct or a kinase-deficient Bcr-Abl construct [12], and GPx-1 protein levels and enzyme activity were determined. The ectopic expression of Bcr-Abl in MDA-MB-231 cells resulted in a significant decrease (0.6-fold) of GPx activity, which was dependent on the presence of an active kinase domain in Bcr-Abl (Fig. 4). Thus, the inhibition of Bcr-Abl with imatinib resulted in increased GPx-1 activity while ectopic expression of the fusion protein reduced GPx-1 activity.

### The effect of imatinib on GPx-1 levels is mediated through the PI3K pathway

**Figure 5:**
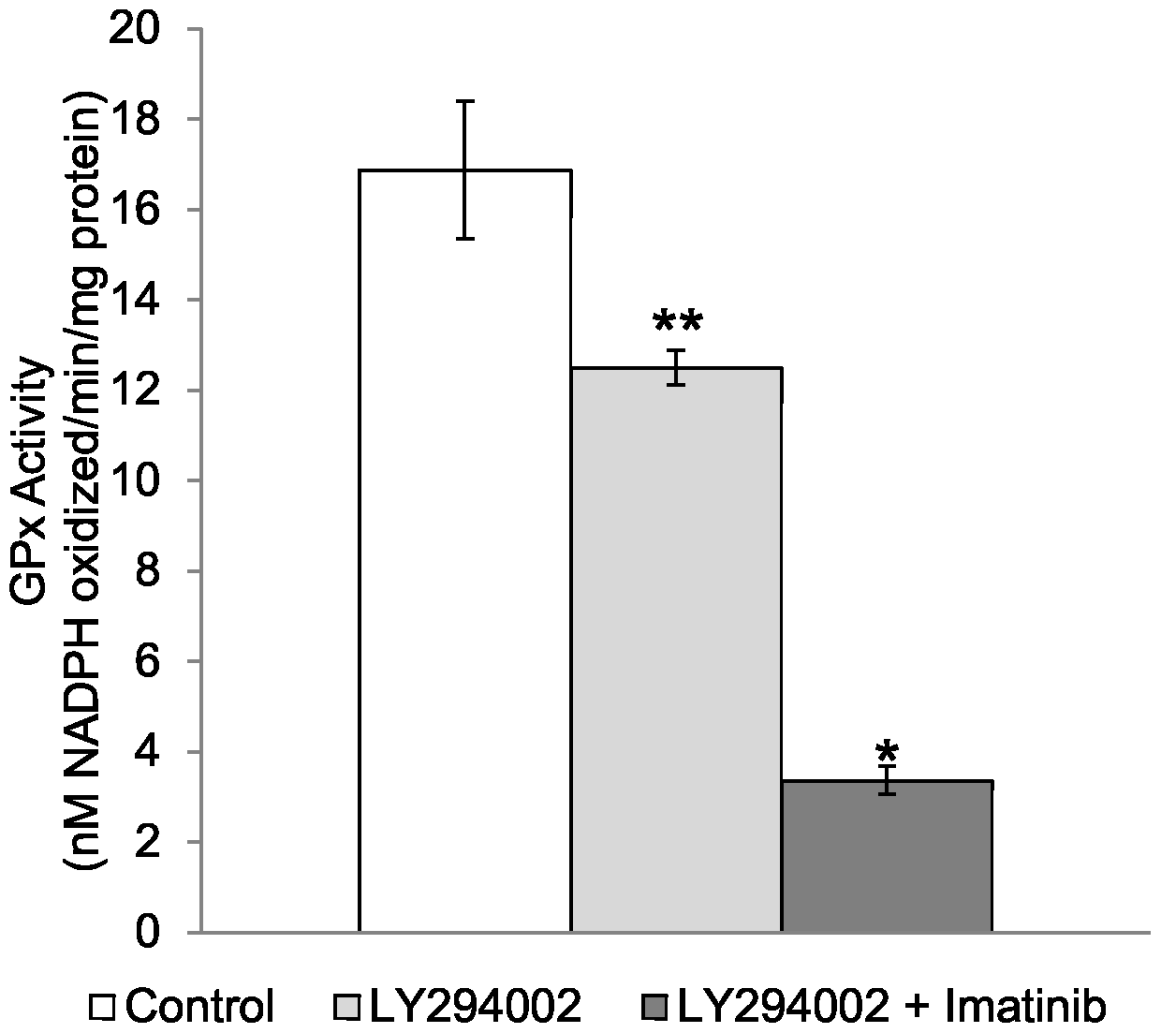
Treatment with LY294002 for 4 days inhibits PI3K phosphorylation activity and decreases GPx activity. The effect of 20 μM LY294002 treatment of KU812a cells on GPx activity levels alone or in combination with 150 nM imatinib is shown. Data presented is derived from three independent experiments. * =  *P*<0.001, ** = *P*<0.01.

Many of the consequences of Bcr-Abl expression are via the phosphotidylinositide 3 kinase (PI3K) pathway [19], [20]. In order to determine if the increase of GPx-1 following inhibition of Bcr-Abl by imatinib required PI3K, the PI3K inhibitor LY294002 (a morpholine derivative of quercetin) was used. Exposure of KU812a cells to 20 μM of LY294002 for 4 days resulted in a small but significant decrease in GPx activity (0.75-fold, Fig. 5). Combinatorial treatment with imatinib and LY294002 not only suppressed the induction observed with imatinib, but dramatically decreased GPx activity. These results indicate that there is likely a signaling pathway that impacts GPx-1 levels which is initiated by Bcr-Abl and requires PI3K in these cells.

### Inhibition of mTOR with rapamycin increases GPx-1 levels and activity

**Figure 6:**
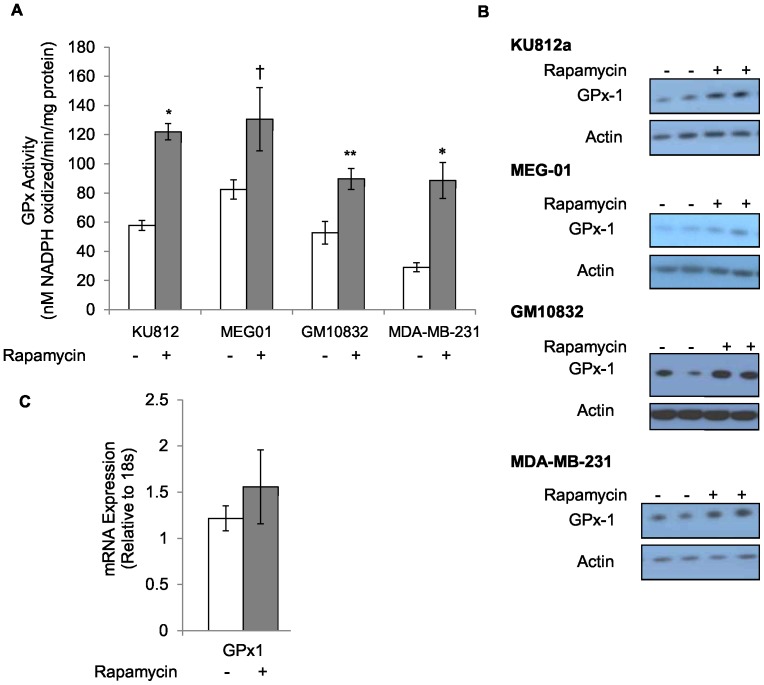
Rapamycin enhances GPx-1 protein and enzyme activity levels in all cell lines investigated. The effect of rapamycin on GPx-1 activity (A) and protein levels (B) in KU812a, MEG-01 GM10832 or MDA-MB-231 is shown. The effect of rapamycin on steady-state GPx-1 transcript levels as determined by RT-qPCR and normalization of GPx-1 Ct values to Ct values for 18s RNA (C). Rapamycin significantly increased protein respectively 3-fold and 1.3-fold in KU812a and MEG-01 (*P* = 0.05). Steady state transcript levels for GPx-1 were unaffected by treatment with rapamycin in KU812a. Data shown is representative of three independent experiments. * = *P*<0.001, ** = *P*<0.01. † = *P*<0.05. Error bars indicate S.D.

The mammalian target of rapamycin (mTOR) is a stress and nutrient response protein that is regulated by the PI3K pathway, one of the pathways induced by Bcr-Abl signal transduction [19], [20]. Given that Bcr-Abl can impact mTOR signaling which in turn affects the translation of many downstream target proteins [21], and Bcr-Abl signaling impacted the translation of GPx-1, the role of mTOR in GPx-1 regulation was investigated. This was accomplished by inhibiting mTOR using rapamycin, a drug that inhibits the mTOR complex I (mTORC1) [22]. Cells were exposed to a dose of 1 ng/ml rapamycin for 72 hours for subsequent studies as these conditions were empirically determined to be cytostatic for all of the lines being examined. KU812a cells exposed to rapamycin exhibited both an increase in GPx-1 activity and protein, as shown in Figs. 6A and 6B, respectively. Similar to what was observed following imatinib exposure, the increase in GPx-1 was not as a result of increased steady-state mRNA levels (Fig. 6C). In contrast to what was seen with imatinib where only Bcr-Abl expressing cells responded to imatinib treatment, exposure to rapamycin also increased GPx-1 activity and levels in both the non-BCR-Abl expressing MDA-MB-231 and GM10832 cells (Fig. 6B).

### Rapamycin increases the levels of another selenoproteins but not other anti-oxidants

**Figure 7:**
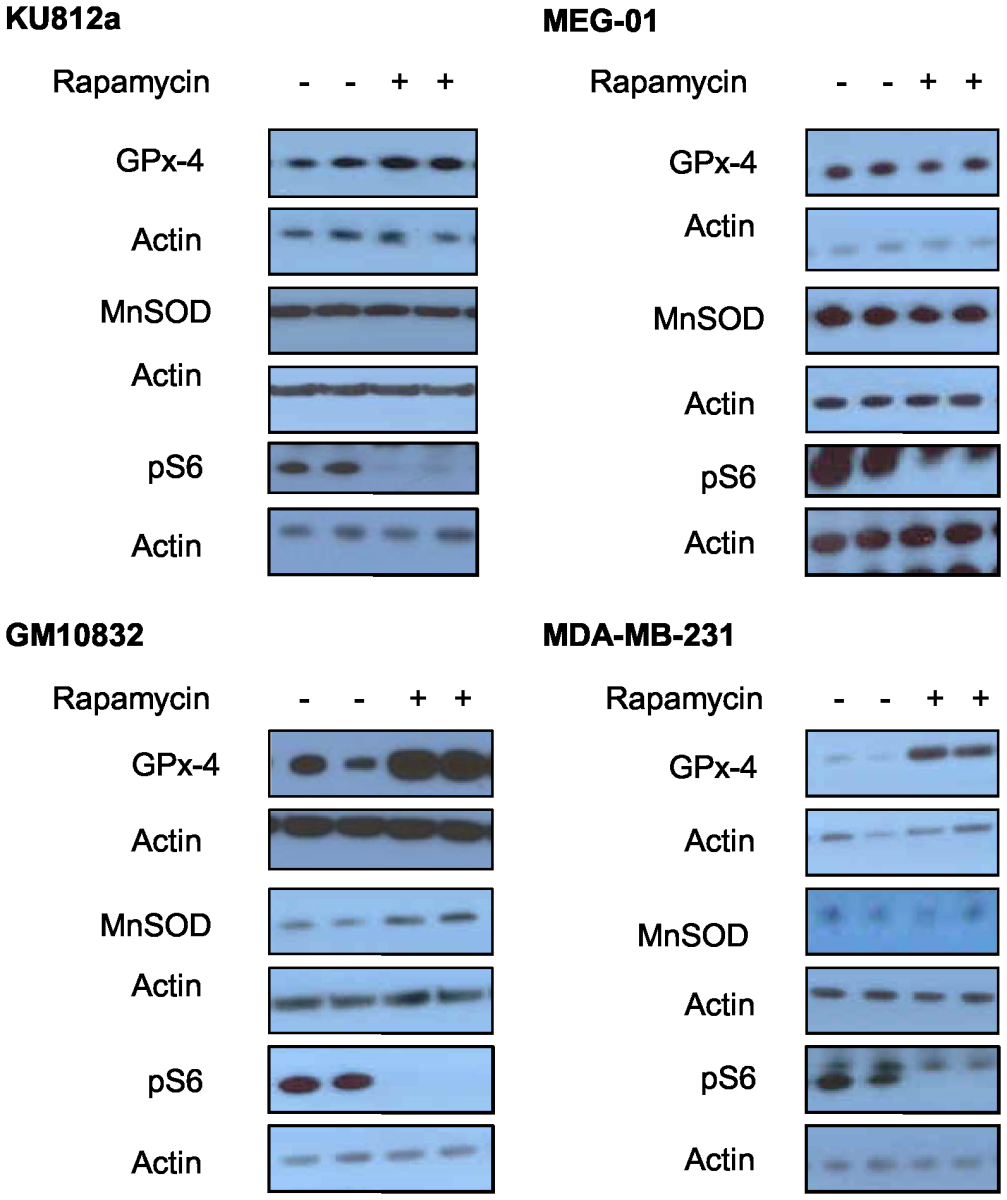
Rapamycin increases levels of GPx-4, MnSOD and pS6 protein levels in cell lines. The effect of 1 ng/mL rapamycin on GPx-4 and MnSOD and pS6 protein levels in KU812a, MEG-01, GM10832 and MDA-MB-231 is shown. GPx-4 by 3-day 1 ng/mL rapamycin treatment in KU812a and MEG-01 (*P* > 0.2), but was increased 3-fold in GM10832 (*P* = 0.05) and 6-fold in MDA-MB-231 cells (*P* = 0.02). The disappearance of pS6 signal following rapamycin treatment indicates inhibition of mTOR. Data shown is representative of three independent experiments.

Since it was determined that exposing cells to rapamycin increased GPx-1 levels, it was next investigated whether the levels of other anti-oxidant proteins were enhanced as well. Manganese superoxide dismutase (MnSOD) is a major protective mitochondrial enzyme that detoxifies superoxide radicals produced during electron transport to the less toxic hydrogen peroxide (H_2_O_2_). Exposure of KU812a, MEG-01, GM10832 or MDA-MB-231 cells to rapamycin did not result in significant induction of MnSOD protein levels (Fig. 7). The effect of rapamycin on two additional selenium-containing anti-oxidant proteins, another member of the GPx family (GPx-4), was also investigated. Induction of GPx-4 protein levels was observed in KU812a, GM10832 and MDA-MB-231 cells, but not in MEG-01 cells (Fig. 7) while levels of TrxR1 remained unchanged in each of the cell lines investigated.

### Rapamycin does not affect the efficiency of the recognition of the UGA codon as selenocysteine

**Figure 8:**
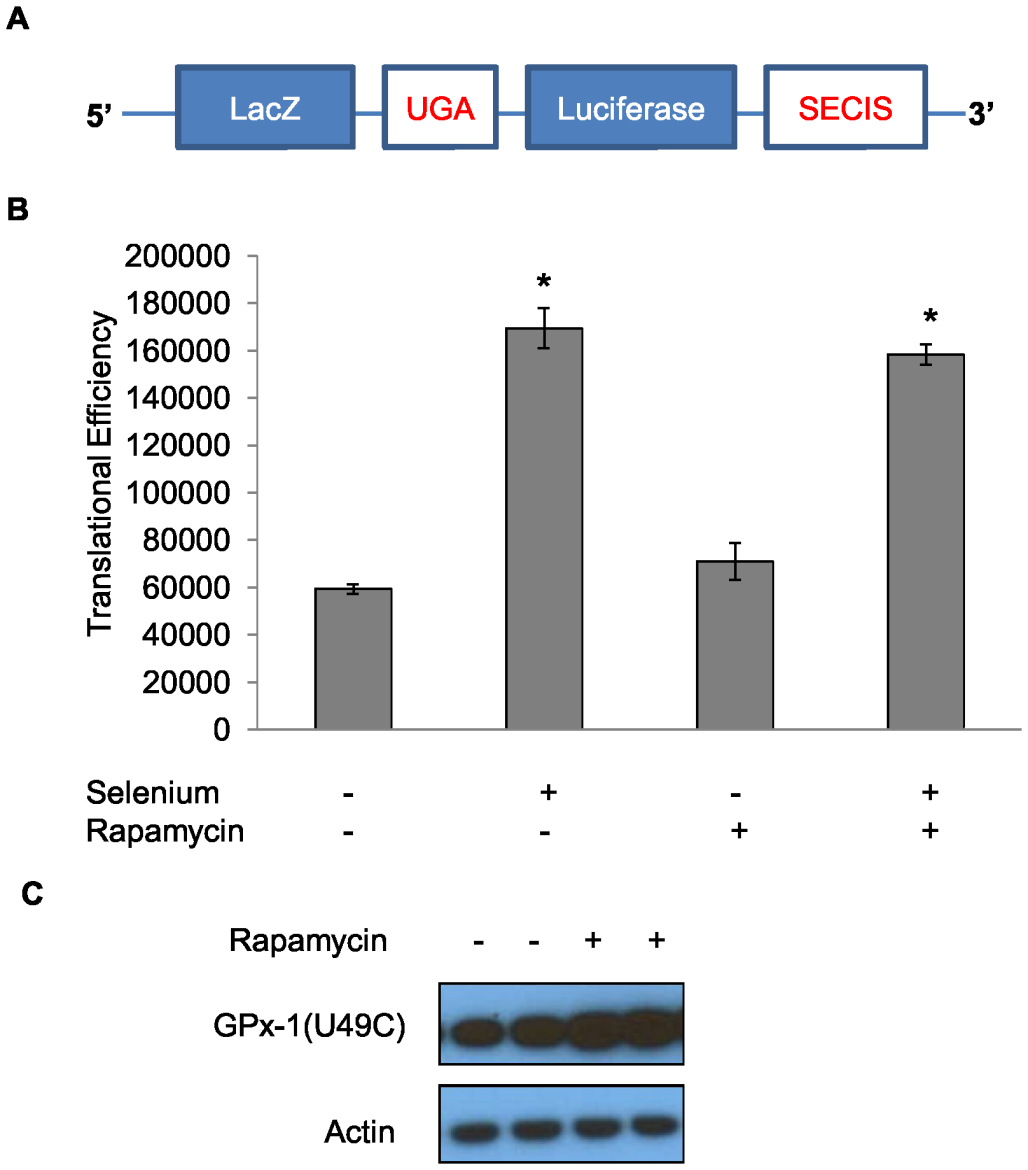
The translational efficiency of UGA readthrough by the GPx-1 SECIS is not enhanced by rapamycin. Diagrammatic representation of the pLNCX-UGA-GPx-1 reporter construct used for quantification of UGA read-through, containing the GPx-1 SECIS element (A). The effect of rapamycin (1 ng/mL), selenium (100 nM), or both on the translational efficiency of UGA readthrough in MDA-MB-231 cells infected with the pLNCX-UGA-GPx1 SECIS reporter construct as assessed by determining luciferase activity; relative luciferase levels were normalized to β-galactosidase levels to calculate translational efficiency (B). SECIS-dependent translational efficiency was not increased following 1 ng/mL rapamycin treatment, but was increased 2.6-fold by incubation of cells containing the reporter construct with selenium. Data shown represents two independent experiments. * = *P*<0.001. Error bars indicate the S.D. The effect of rapamycin on U49C GPx-1 levels was determined by immunoblotting for GPx-1 by western blot analysis (C).

Since the inhibition of mTOR with rapamycin resulted in the elevation of the levels of another selenoprotein, GPx-4, it was investigated whether rapamycin enhanced the decoding of the in-frame UGA codon in selenoprotein mRNAs as selenocysteine. To accomplish this, a reporter construct was used that contains the coding sequence for β-galactosidase (β ˜gal) fused to the open reading frame of the LacZ gene that encodes luciferase (luc) separated by an in-frame UGA codon [13], see Fig. 8A for a schematic representation of the construct. In the absence of a SECIS element, only β ˜gal is generated while the inclusion of the GPx-1 SECIS element 3′ of the coding sequence of the fusion protein permits UGA read-through with the ratio of measured luc to β—gal activities being a measure of the efficiency of UGA recognition as selenocysteine. A derivative retroviral reporter plasmid was generated from an existing reporter containing the SECIS element from human GPx-1 [14] and used to infect MDA-MB-231 cells that were treated with rapamycin. These cells were also treated with low levels of selenium previously shown to stimulate translation of the fusion protein as a positive control for the enhancement of UGA read-through. SECIS-dependent translational efficiency was not increased following 1 ng/mL rapamycin treatment, but was increased 2.6-fold by selenium exposure, indicating that the increase in GPx-1 levels achieved with rapamycin was not likely due to enhanced UGA translation as selenocysteine (Fig. 8B). In order to obtain additional evidence that the stimulation of GPx-1 levels by rapamycin was not due to the enhanced decoding of UGA as selenocysteine, a derivative GPx-1 expression construct was generated in which the UGA codon at position 49 was converted to a cysteine triplet, referred to as U49C. GPx-1 generated from this construct was translated at high efficiency due to the relative inefficiency of recognizing UGA as selenocysteine. This construct was transfected into MCF-7 cells that do not produce datectable levels of GPx-1 [17], [23]. As seen in Fig. 8C, exposure of U49C-expressing cells to 1 ng/mL rapamycin for 3 days resulted in an induction of the selenocysteine-containing GPx-1 protein to a level comparable to the level of stimulation observed for endogenous, UGA-encoding GPx-1 (Fig. 6). Collectively, the use of a selenocysteine incorporation reporter construct and the U49C version of GPx-1 provide evidence that the stimulation of GPx-1 and GPx-4 by rapamycin is not due to the enhanced translational efficiency of UGA codons.

## Discussion

This study was initiated because our previous results indicated a higher level of GPx activity in mononuclear cells obtained from patients post-imatinib treatment [11]. Using two different CML-derived cell lines, we established that exposure of these cells to imatinib increased GPx activity and GPx-1 levels, this being consistent with what was observed by examining the patient samples. It was also determined that the increase in GPx-1 levels was not due to enhanced GPx-1 transcription and that the effects of imatinib required the expression of the Bcr-Abl fusion protein. Increased expression of GPx-1 in Bcr-Abl expressing clones exposed to imatinib might be expected to have significant impact on the clinical outcome of CML patients. Elevated GPx-1 activity has been shown to prevent apoptosis [24] and reduce the levels of DNA damage [25], [26], as well as alter reactive oxygen-mediated signaling pathways implicated in growth control and carcinogenesis [7]. These data therefore raise the possibility that GPx-1 induction by Bcr-Abl inhibition may have a detrimental effect on the efficacy of treatment, as well as the efficacy of approaches to treat CML once resistance to front line therapy has failed.

The PI3K/AKT/mTOR pathway is frequently activated in a wide range of malignancies, and it is deregulated in response to Bcr-Abl activation as well as in the development of resistance to Bcr-Abl inhibitors [19], [27]–[29]. This pathway also appears to be involved in the regulation of GPx-1 as the inhibition of PI3K with LY294002 resulted in an approximately 25% reduction in GPx-1 activity and had a dramatic effect when co-administered with imatinib, resulting in a better than 5-fold reduction in GPx-1 activity. Imatinib and LY294002 have been shown to act synergistically in inducing apoptosis and autophagy in Bcr-Abl expressing leukemia cells [30]. The observation that combining LY294002 with imatinib had a dramatic effect in reducing GPx activity was therefore surprising as imatinib alone increased GPx-1 levels and activity. These observations lend support to the complexity of the pathways affected by these inhibitors as also indicated by the less than desired results obtained by combining inhibitors of Bcr-Abl and mTOR in the clinical management of both CML and gastrointestinal stromal tumors that harbor activating mutations in receptor tyrosine kinases [29], [31]. However, the link between the relationship of the Bcr-Abl and PI3K pathways, the observation that rapamycin could reduce ROS levels in Bcr-Abl transformed cells [20], and the ability of mTOR to affect protein synthesis prompted us to assess whether the inhibition of mTOR with rapamycin also affected the translation of GPx-1.

The induction of GPx-1 levels and activity achieved by the inhibition of mTOR with rapamycin was post-transcriptional and did not require expression of Bcr-Abl in exposed cells. The translation of GPx-1, like the other members of the selenocysteine-containing family of proteins, require a number of dedicated translation factors in order to decode UGA as selenocysteine, which is a relatively inefficient process [32]–[34]. In addition, the induction of another selenocysteine protein of the GPx-1 family, GPx-4, by rapamycin in some of the examined cell lines raised the possibility that imatinib and rapamycin were increasing GPx-1 levels by increasing UGA readthrough. However, this is unlikely as rapamycin treatment was unable to stimulate UGA readthrough using a reporter construct and the induction of GPx-1 by rapamycin occurred to similar extent whether GPx-1 contained a UGA or a cysteine encoding triplet at position 49. Regulation of protein synthesis by mTOR is still an area under investigation. A recent analysis of the translation of proteins affected by another mTOR inhibitor, Torin1, by high resolution ribosome profiling revealed that the inhibition of mTOR resulted in the suppressed translation of the vast majority of transcripts, and more severely repressed the translation of transcripts with 5’ terminal oligopyrimidine (TOP) motifs; however, the translation of both GPx-1 and GPx-4 were enhanced in Torin1 treated mouse embryo fibroblasts [35]. Rapamycin has been shown to increase both the total levels of another anti-oxidant enzyme, MnSOD as well as its post-translational modification by acetylation [36]. Deacetylation of MnSOD occurs in the mitochondria by the Sirt3 deacetylase, a member of the sirtuin family of proteins [37]. Although the levels of MnSOD did not increase when the cells we examined were treated with rapamycin, it remains interesting that both GPx-1 and GPx-4, reside in the mitochondria [38]–[41] and are modified by acetylation in that organelle [8], [9], [42], [43]. Future studies will be directed towards pursuing the possibility that post-translational modification of GPx proteins by acetylation contributes to the effects of imatinib and rapamycin described in this manuscript.
